# Chronic gamma-hydroxybutyric-acid use followed by gamma-hydroxybutyric-acid withdrawal mimic schizophrenia: a case report

**DOI:** 10.4076/1757-1626-2-7520

**Published:** 2009-07-10

**Authors:** Prometheas Constantinides, Philippe Vincent

**Affiliations:** 1 Department of Psychiatry, Louis-H. Lafontaine Hospital7401 Hochelaga, Montreal, QuebecCanada; 2 Department of Pharmacy, Louis-H. Lafontaine Hospital7401 Hochelaga, Montreal, QuebecCanada

## Abstract

**Introduction:**

Gamma-hydroxybutyric-acid is a potentially addictive drug known for its use in “rave” parties. Users have described heightened sexual drive, sensuality and emotional warmth. Its euphoric, sedative and anxiolytic-like properties are also sought by frequent users. Abrupt gamma-hydroxybutyric-acid withdrawal can rapidly cause tremor, autonomic dysfunction and anxiety, and may later culminate in severe confusion, delirium, auditory, visual or tactile hallucinations, or even death.

**Case presentation:**

A 23-year-old woman presented to the emergency room with paranoid delusions and auditory hallucinations. Her psychiatric history included two brief psychotic episodes induced by amphetamines and marijuana. In the last six months, she had demonstrated bizarre behaviour, had been more isolated and apathetic, and unable to take care of daily chores. The patient reported occasional use of gamma-hydroxybutyric-acid, but her initial accounts of drug use were contradictory. Since the toxicology urine screen was negative, a schizophrenic disorder was initially suspected and an antipsychotic medication was prescribed. A few hours after her admission, signs of autonomic dysfunction (tachycardia and hypertension) appeared, lasting 24 hours. Severe agitation and confusion were also present. Restraints and a cumulative dose of 7 mg lorazepam were used to stabilize her. The confusion resolved in less than 72 hours. The patient then revealed that she had been using gamma-hydroxybutyric-acid daily for the last six months as self-medication to treat insomnia and anxiety, before stopping it abruptly 24 hours prior to her visit.

**Conclusions:**

In our opinion, this original case illustrates the importance of considering gamma-hydroxybutyric-acid withdrawal delirium in the differential diagnosis of a first-break psychosis. In this case, the effects of chronic GHB use were incorrectly identified as the negative symptoms of schizophrenia prodrome. Likewise, severe gamma-hydroxybutyric-acid withdrawal syndrome was initially mistaken for acute positive symptoms of schizophrenia, until autonomic dysfunction manifested itself more clearly.

## Introduction

Gamma-hydroxybutyric-acid (GHB) is a potentially addictive drug used in “rave” parties to heighten sexual drive, sensuality and emotional warmth. Its euphoric, sedative and anxiolytic-like properties, and ability to relieve inhibitions, are also sought by frequent users [1]. Most of the desired effects are mediated by a direct stimulation of GABA_B_ receptors and auto receptors in the brain, and also by its metabolism to GABA in presynaptic neurons. This pathway is also that of GHB intoxication. The drug’s addictive properties are mediated by decreased GABA release in the ventral tegmental area which causes an inhibition of dopaminergic neurons firing in the nucleus accumbens and frontal cortex [1]. Regular administration of GHB causes down regulation of these GABA receptors, leading to drug dependence as a means to maintain homeostasis. Abrupt GHB withdrawal will cause imbalance between under-stimulated GABA neurons and stimulatory input in the ventral tegmental area. This will cause withdrawal symptoms - tremor, autonomic dysfunction and anxiety - to appear as soon as one to 24 hrs after the last dose, resulting in drug craving. Although mild at presentation, symptoms may culminate in one to seven days to severe confusion, delirium, auditory, visual or tactile hallucinations, or even death [2].

## Case presentation

We report the case of a 23-year-old French Canadian woman who presented at the Emergency Room in summer 2006 with paranoid delusions and auditory hallucinations. Her previous history included two brief psychotic episodes induced by substances (amphetamines and marijuana). During these previous psychotic episodes, she exhibited ideas of reference and visual hallucinations which had responded well to olanzapine, an atypical antipsychotic.

In the last six months, she had shown bizarre behaviour, had been more isolated and apathetic, and unable to participate in daily chores. Her motivation had declined. She had gradually withdrawn from significant social relationships and stopped working. She described feeling anxious on a regular basis with no apparent reason. This was confirmed by family members accompanying her. The patient reported occasional use of GHB, but as her initial accounts of drug use were contradictory, the product used could not be positively identified and frequency of use remained unknown at the time.

Upon admission, the patient appeared perplexed and entertained the unsubstantiated fear that someone would try to kill her. She was under the impression that her whole entourage was speaking ill of her behind her back. She was whispering for fear of being heard, and attacked. She reported not having slept in days. She also mentioned visual hallucinations, claiming to have seen tigers in her apartment. The toxicology urine screen was negative. No evidence of depressive or manic symptoms was found. Patient history and physical examination indicated no general medical condition.

The initial suspicion of a schizophrenic disorder was in accordance with the terms of this diagnosis as described in the DSM-IV: The patient presented an acute psychotic break (hallucinations and delusions, part of Criterion A of the DSM-IV diagnosis), preceded by a prodrome of negative symptoms (prolonged apathy and lack of motivation, part of Criterion A), lasting six months (Criterion C), with a significant decline in social and occupational functioning (Criterion B). Mood disorders were excluded (Criterion D). Substance-induced and general medical condition-induced psychoses were excluded as unlikely (Criterion E). The patient was thus prescribed olanzapine 10 mg at bed time.

**Figure 1.**
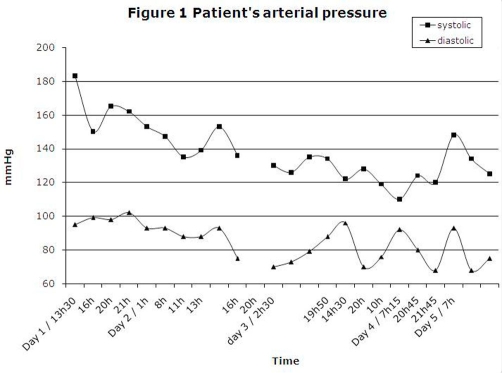
Patient’s arterial pressure.

**Figure 2.**
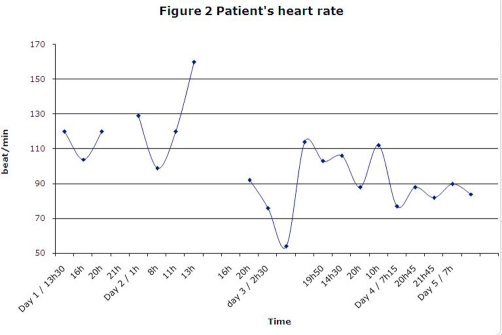
Patient’s heart rate.

A few hours after her admission, she started presenting labile vital signs (Figure 1 and 2): tachycardia (range 160-120 beat/min) and hypertension (range 183/95-139/88 mmHg) that lasted for 24 hours. The patient’s temperature was not taken. She also suffered from severe agitation and anxiety, showed disorganized speech and behaviour, had insomnia, and possibly visual hallucinations. Restraints and confinement had to be used to protect the patient from injury, and a cumulative dose of 7 mg lorazepam p.o. over 24 hours was necessary to stabilize her. At this point, we suspected a withdrawal delirium, either from ethanol or GHB because of the autonomic system instability. Olanzapine was discontinued (of which she had received a single dose). Apart from lorazepam, she did not require any further drug treatment. Her confusion resolved in less than 72 hours. She suffered from a complete amnesia of the episode.

At this point, the patient revealed that she had been using GHB daily for the last six months as a self-medication to treat insomnia and anxiety. She denied using any other drug or medication during that time, including alcohol. She had developed a dependence to GHB as evidenced by increased anxiety, tremor and insomnia whenever she skipped a dose, and also by the increasing amount of time spent seeking and using GHB. She also admitted to often being very sedated while using GHB, a frequent state which resulted in a general lack of motivation and interest. One day prior to her admission, the patient had decided to put an end to her GHB use, an abrupt interruption which provoked the onset of intense anxiety and paranoid delusions within 18 hours, followed by disorganisation, and auditory and visual hallucinations within 24 hours.

## Conclusions

In our opinion, this original case illustrates the importance of considering GHB withdrawal delirium in the differential diagnosis of a first break psychosis. In this case, chronic GHB use was incorrectly perceived as a schizophrenic prodrome characterized mainly by negative symptoms (Table 1). Likewise, a severe GHB withdrawal syndrome was initially mistaken for acute positive symptoms of schizophrenia, i.e. hallucinations and delusions, until autonomic dysfunction manifested itself more clearly (Table 2).

**Table 1.** Overlap between negative symptoms of schizophrenia and GHB chronic use

Symptoms of Chronic Use of GHB	Negative Symptoms of Schizophrenia
Lack of volition and drive	
Poverty of speech	
Loss of feeling	
Social withdrawal	

**Table 2.** Overlap between positive symptoms of schizophrenia and GHB withdrawal delirium

GHB withdrawal delirium	Symptoms of Schizophrenia
Disorganized Speech	
Disorganized Behavior, Agitation	
Delusions	
Hallucinations	
Autonomic Dysfunction	

In a literature search, no other reports of this phenomenon were found. Since GHB is not usually part of routine toxicology screens, it is important to consider GHB use and GHB withdrawal delirium in the differential diagnosis of psychosis, especially since autonomic dysfunction is often milder in GHB withdrawal compared to that of ethanol [3], and since the treatment is different. Obtaining a detailed history from the patient and family members and keeping regular track of the vital signs may help make the proper diagnosis.

The treatment of a GHB withdrawal delirium calls for a treatment similar that of to ethanol or benzodiazepine withdrawal, mainly: admission, supportive treatment, and a tapering regimen of benzodiazepines. Unless a delirium is present, antipsychotic drugs are not indicated in GHB withdrawal^3^, whereas in schizophrenia, they are the cornerstone of the treatment.

## Abbreviations

DSM IV: Diagnostic and Statistical Manual of Mental Disorders, 4th Edition
GABA: gamma-aminobutyric acid
GHB: gamma-hydroxybutyric-acid
